# Suppression of Gq and PLC gene expression has a small effect on quantum bumps in vivo in *Periplaneta americana*

**DOI:** 10.1007/s00359-020-01417-7

**Published:** 2020-04-13

**Authors:** Irina I. Ignatova, Andrew S. French, Päivi H. Torkkeli, Hongxia Liu, Roman V. Frolov

**Affiliations:** 1 Nano and Molecular Systems Research Unit, University of Oulu, Oulu, Finland; 2 Department of Physiology and Biophysics, Dalhousie University, P.O. BOX 15000, Halifax, NS B3H 4R2 Canada

**Keywords:** Insect photoreceptor, Gq protein, PLC, Phototransduction

## Abstract

Visual signal transmission by *Drosophila melanogaster* photoreceptors is mediated by a Gq protein that activates a phospholipase C (PLC). Mutations and deficiencies in expression of either of these proteins cause severe defects in phototransduction. Here we investigated whether these proteins are also involved in the cockroach, *Periplaneta americana*, phototransduction by silencing Gq α-subunit (Gqα) and phosphoinositide-specific phospholipase C (PLC) by RNA interference and observing responses to single photons (quantum bumps, QB). We found (1) non-specific decreases in membrane resistance, membrane capacitance and absolute sensitivity in the photoreceptors of both Gqα and PLC knockdowns, and (2) small changes in QB statistics. Despite significant decreases in expressions of Gq and PLC mRNA, the changes in QB properties were surprisingly modest, with mean latencies increasing by ~ 10%, and without significant decrease in their amplitudes. To better understand our results, we used a mathematical model of the phototransduction cascade. By modifying the Gq and PLC abundances, and diffusion rates for Gq, we found that QB latencies and amplitudes deteriorated noticeably only after large decreases in the protein levels, especially when Gq diffusion was slow. Also, reduction in Gq but not PLC lowered quantum efficiency. These results suggest that expression of the proteins may be redundant.

## Introduction

The microvillus of an insect photoreceptor is a highly organized compartment containing molecular machinery to reliably and repeatedly generate quantum bumps (QB), the electrical responses to absorption of single photons. A typical microvillus in a photoreceptor of a fly compound eye is ~ 1 µm long and ~ 55 nm wide, while its membrane contains ~ 1000 rhodopsin molecules (~ 65% of total membrane protein, estimated from Kumar and Ready (1995), Paulsen and Schwemer (1979) and Schwemer and Henning (1984)). The actual concentrations of other molecules involved in phototransduction in the *D. melanogaster* microvillus are, to our knowledge, not yet determined, but they have been estimated to contain ~ 100 trimeric Gq proteins, ~ 100 phospholipase C (PLC) molecules, ~ 3000 phosphatidylinositol 4,5-bisphosphate (PIP_2_) molecules that fuel the phototransduction reaction, ~ 25 tetrameric cationic TRP and TRPL channels that generate the electrical responses, and ~ 100 protein kinase C (PKC) molecules that are involved in the response termination (Huber et al. 1996; Nikolic et al. 2010).

The TRP and TRPL channels, PLCs, and PKCs are tethered to the central axial actin filament via links to scaffolding protein INAD and myosin III (NINAC), which render them effectively immobile (Hardie and Raghu 2001). NINAC also harbors calmodulin and arrestin, the soluble regulators of the onset and termination of the phototransduction cascade (Scott and Zuker 1998). As rhodopsin is also considered essentially immobile (Nikolic et al. 2010), signal propagation from the activated receptor (metarhodopsin) to PLC depends on the diffusional sliding of Gq proteins below the plane of the membrane, whereas the catalytic activity of PLC depends on the free diffusion of PIP_2_ within the membrane.

The main events of the stochastic phototransduction cascade in *D. melanogaster* photoreceptors involve activation of a rhodopsin molecule by a photon, a random encounter of the freely diffusing Gq protein with the metarhodopsin; Gq protein binding and conformational change; replacement of GDP with GTP on the α-subunit; dissociation of Gqαβγ to Gqα and Gqβγ and their release from metarhodopsin; free diffusion of Gqα until its encounter and binding with PLC, initiation of enzymatic activity by Gqα-PLC; sequential breakdown of multiple molecules of PIP_2_ with release of diacylglycerol (DAG), inositol trisphosphate (IP_3_) and H^+^; and, finally, mechano-chemical gating of the TRP and TRPL channels (Hardie and Juusola 2015). Both the activity of PLC and channel gating are regulated by the influx of Ca^2+^, which is also crucial for the timely termination of the quantum bump and inactivation of the metarhodopsin (Hardie and Juusola 2015).

The cascade amplifies at two stages: first, a single metarhodopsin continues to activate multiple Gq proteins until quenched by arrestin; second, each Gqα–PLC complex breaks down numerous PIP_2_ molecules until a threshold is reached for the all-or-nothing opening of TRP/TRPL channels, which may require splitting hundreds of PIP_2_ molecules. In case of a single photon absorption event, the two stages of amplification must overlap substantially because as metarhodopsin continues activating new Gq proteins, the Gqα–PLC complexes formed earlier are already operating. The number of active Gqα–PLC complexes cannot exceed the number of activated Gq proteins; each Gqα–PLC remains active until the GTP inside the Gqα is hydrolyzed as a result of interaction with the PLC, which also serves as a GTPase-activating protein. However, this normally happens after a substantial delay comparable with the entire latent period (Nikolic et al. 2010).

The main question of the present study was why so many Gq and PLC proteins appear to be expressed in the microvillus if only a small fraction of them are activated during a phototransduction event. Rhodopsin and PIP_2_ are even more abundant in the microvillus but high density of the photon catcher rhodopsin is needed for high absolute sensitivity, whereas PIP_2_ needs to be actively replenished. PLC levels are similar to those of Gq and these two molecules interact with 1:1 stoichiometry, PLC directly downstream of Gq. Thus, changes in Gq and PLC concentrations should cause similar effects. Indeed, studies of *D. melanogaster* mutants with very low levels of Gq and PLC demonstrated similar phenotypes characterized by dramatically reduced quantum bump efficiencies, increased latencies and decreased quantum bump amplitudes (Hardie et al. 2002; Scott and Zuker 1998).

Here we investigated whether knockdown of Gqα and PLC in the *Periplaneta americana* retina would change the statistical properties of quantum bumps. We knocked down Gqα and PLC genes using RNA interference (RNAi) and recorded quantum bumps in vivo using the intracellular recording technique. We then developed a stochastic model of *P. americana* quantum bumps based on a previously created model in *D. melanogaster* (Nikolic et al. 2010).

## Methods

American cockroaches, *Periplaneta americana* (Linnaeus), were reared locally at 25 °C under reversed 12 h:12 h illumination conditions but with shelter from light always available. Only healthy adult male cockroaches without physical injuries and behaviorally agile were used in experiments.

### RNA interference

The putative *P. americana* Gqα and phosphoinositide-specific PLC were identified from the retinal transcriptome as described for other cockroach genes previously in detail (French 2012; French et al. 2015). Long double-stranded RNA (540 bp for Gqα and 683 bp for PLC) was synthesized using similar methods as described earlier (French et al. 2015). Reverse transcription was performed using total RNA extracted from cockroach retinas and oligo-d(T)23VN primers with ProtoScript II reverse transcription (New England Biolabs, Whitby, Ontario, Canada). The reverse transcription product was used in PCRs to amplify the template DNAs using Q5 High-Fidelity DNA Polymerase (New England Biolabs). dsRNA was synthesized with the MEGAscript RNAi kit (Thermo Fisher Scientific, Waltham MA). Cockroaches were anaesthetised with CO_2_ and Hamilton 5-µL syringe with a beveled needle attached was used to inject 1 µL of the dsRNA (4 µg/µL injection buffer that contained 0.1 µM Na phosphate buffer and 5 µM KCl) into the head. Control animals were injected with 1 µL of the injection buffer. 8–10 cockroaches were used for each gene and for the control. After the injections, animals were maintained in separate cages but otherwise under identical conditions to normal.

### Relative RNA expression

Retinas were collected from 7 to 9 cockroaches 21 days after dsRNA injection for quantitative real-time PCR (RT-qPCR) analysis as described earlier (French et al. 2015). An RNeasy Plus mini kit (Qiagen, Valencia, CA, USA) was used to extract the total RNA and the amount of mRNA was evaluated using an Experion RNA Analysis Kit (Bio-Rad, Mississauga, Ontario, Canada) after treatment with RNase-free DNase I (Ambion). 50 ng of total RNA was used for first-strand cDNA synthesis with ProtoScript II reverse transcriptase (New England BioLabs). Quantitative PCR was performed using GoTaq qPCR Master Mix (Promega, Madison, WI, USA) on a CFX Connect real-time PCR detection system (Bio-Rad). The gene-specific primers are shown in Table 1. Gene expression levels and PCR efficiency were calculated using CFX Manager software (Bio-Rad). Amplification efficiencies of the primers were determined using serially diluted cDNA samples. All PCR runs were performed in triplicate and the relative expression levels were calculated using CFX Manager software.

**Table 1:** Primers used for quantitative PCR analysis

Gene and direction	Sequence	Amplicon (bp)	Efficiency (%)
*gqα* Forward	CCAAGAGTGCTATGATAGGAGACG	131	92.12
*gqα* Reverse	GCGCTCTCGCTCTGAGAATG		
*plc* Forward	TGATGGATCAGGTGCAGGTG	146	90.42
*plc* Reverse	TCTGGTAGCCCTTCTCTGAGC		
*actin* Forward	GTACGTTGCTATCCAGGCTGTG	158	85.60
*actin* Reverse	AATCGCGACCAGCCAGATC		
*gapdh* Forward	GTGTTCCTGTTCCCAATGTTTC	134	89.51
*gadph* Reverse	TTCAGTGTAGTCCAAGATGCC		

### Electrophysiology

Cockroaches were anaesthetised with CO_2_ and immobilized in a plastic pipette with the upper body and the head protruding. To stop muscle movements, deep incisions were made in the middle of the frons (vertical) and in the fronto-clypeal suture (horizontal). Wounds were sealed with wax. Jaw movements were restrained by sealing the mouth with wax. All palps and the left antenna were also immobilized with wax. The reference electrode (Ag/AgCl wire) was inserted through a small cut in the left antenna and fixed with wax. The right antenna was either immobilized with wax or, during prolonged experiments, left intact to monitor the well-being of the animal. A small hole for the recording electrode was made in the dorsal part of the left eye and immediately sealed with silicon grease.

Microelectrodes were made from borosilicate glass (Harvard Apparatus, Cambridge, MA, USA) using a laser puller (P-2000; Sutter Instrument, Novato, CA, USA), filled with 2 M KCl solution, pH 6.84 with potassium phosphate buffer, and had resistance of 100–130 MΩ.

Microelectrodes were inserted into the retina using a micromanipulator (SMX-model, Sensapex Oy, Oulu, Finland). The light source, mounted on a cardan arm, was aligned with the photoreceptor’s optical axis. The electrode capacitance was compensated, and signals were amplified with a single-electrode intracellular amplifier (SEC-05L; NPI, Germany) and recorded using custom Matlab (Natick, MA, USA) software.

Membrane capacitance and resistance were measured in the current-clamp mode using voltage responses to step current injections, usually a hyperpolarizing 0.5 s pulse of − 0.25 nA. Membrane resistance was determined as the amplitude of the voltage response after membrane charging was complete divided by the injected current. Capacitance was derived by dividing the membrane time constant, obtained by fitting the rising phase of the voltage response with a single exponential function, by the membrane resistance value.

1 ms flashes of green light emitted by a LED with a peak at 525 nm were used to evoke quantum bumps. Stimulus intensity was attenuated with a series of neutral density (ND) filters (Kodak, New York, NY, USA). In absolute terms, the light intensity was about 2.2 × 10^11^ photons cm^−2^ s^−1^ for the green LED (525 nm) at the light guide end for the light level at which the median absolute sensitivity in control was found (see “Results”). Recordings were performed from green-sensitive photoreceptors at room temperature (22–24 °C).

### Modelling the *P. americana* single-photon responses to light

To simulate the *P. americana* quantum bumps (Fig. 5a), we used one of the models previously created for *D. melanogaster* (Nikolic et al. 2010) [for another model, see Song and Juusola (2017), Song et al. (2012)].

#### Overview of the model

Below we provide a brief overview of the model by Nikolic et al. (2010), with key equations and with emphasis on the equations and parameters we changed in our simulations. It should be noted that this overview is not comprehensive; the reader is encouraged to read the original article. The model consists of four modules: metarhodopsin deactivation, cascade amplification, the TRP channel model, and the currents model modules.

Upon conversion of a rhodopsin molecule into metarhodopsin, the rate of generating Gqα (G* in equations below) can be expressed as

1 $${\nu }_{1}\left(t\right)=\frac{1}{{\tau }_{\mathrm{c}\mathrm{o}\mathrm{l}\mathrm{l}}\left(t\right)+{\tau }_{\mathrm{G}\mathrm{D}\mathrm{P}}},$$

where *τ*_GDP_ is time for GDP–GTP exchange, and metarhodopsin–Gq protein collision time constant *τ*_coll_:

2 $${\tau }_{\mathrm{c}\mathrm{o}\mathrm{l}\mathrm{l}}\left(t\right)=\frac{{S}_{\mathrm{m}\mathrm{v}}}{{\alpha }_{1}{D}_{\mathrm{G}}\left[{G}_{\mathrm{t}\mathrm{o}\mathrm{t}}-{G}^{\mathrm{*}}-{GPLC}^{*}\right]},$$

where *S*_mv_ is the microvillus membrane area, *α*_1_ the metarhodopsin–Gq protein collision factor, *D*_G_ the diffusion rate for trimer Gq proteins, *G*_tot_ the total number of Gq proteins, and GPLC* the activated PLC. It is presumed that metarhodopsin is essentially immobile.

The change in the number of activated Gq proteins can be expressed as

3 $$\frac{d{G}^{*}}{dt}={\nu }_{1}\left(t\right)\left[{M}^{*}\left(t\right)*\frac{\partial }{\partial t}{f}_{\mathrm{a}\mathrm{c}\mathrm{t}}\left(t, {\tau }_{1}\right)\right]- {\nu }_{2}\left(t\right){G}^{*}\left(t\right),$$

where metarhodopsin availability function is convolved with the activation function ${f}_{\mathrm{a}\mathrm{c}\mathrm{t}}\left(t,\tau \right)=1-{e}^{-t/\tau }$, describing the release of activated Gq protein after a delay. *ν*_2_(*t*) is the rate of GPLC* forming:

4 $${\nu }_{2}\left(t\right)={\alpha }_{2}{D}_{{G}^{*}}\frac{{PLC}_{\mathrm{t}\mathrm{o}\mathrm{t}}}{{S}_{\mathrm{m}\mathrm{v}}{\left[1+\sqrt{G{PLC}^{*}(t)/\pi }\right]}^{2}},$$

where *α*_2_ the Gq–PLC collision factor, *D*_G*_ the diffusion rate for the activated Gq proteins, *PLC*_tot_ the total number of PLC proteins.

The kinetics of Gqα-PLC is given by

5 $$\frac{d{\mathrm{G}\mathrm{P}\mathrm{L}\mathrm{C}}^{*}}{dt}={\nu }_{2}\left(t\right){G}^{*}\left(t\right)-\frac{{\mathrm{G}\mathrm{P}\mathrm{L}\mathrm{C}}^{*}\left(t\right)}{{\tau }_{\mathrm{P}}\left({\mathrm{C}\mathrm{a}}^{2+}\right)},$$

The time constant *τ*_P_ describes the decay of the Gqα–PLC complex. This process is calcium dependent, but also proceeds in the dark due to the intrinsic autocatalytic GTPase activity:

6 $${\tau }_{\mathrm{P}}\left({\mathrm{C}\mathrm{a}}^{2+}\right)={\tau }_{\mathrm{P}, \mathrm{d}\mathrm{a}\mathrm{r}\mathrm{k}}{e}^{-{\beta }_{2}{A}_{\mathrm{G}\mathrm{A}\mathrm{P}}\left({\mathrm{C}\mathrm{a}}^{2+}\right)},$$

where *τ*_P,dark_ is the basal GTPase activity at rest, *A*_GAP_ a function describing the calcium dependence of the GTPase activity and *β*_4,_ an activation constant for the action of GAP on PLC.

Activated PLC produces DAG at the rate:

7 $${\nu }_{3}\left(t,{\mathrm{C}\mathrm{a}}^{2+}\right)=\frac{1}{{\tau }_{\mathrm{c}\mathrm{o}\mathrm{l}\mathrm{l},{\mathrm{P}\mathrm{I}\mathrm{P}}_{2}}\left(t\right)+{\tau }_{\mathrm{r}\mathrm{e}\mathrm{a}\mathrm{c}\mathrm{t},\mathrm{P}\mathrm{L}\mathrm{C}}\left({\mathrm{C}\mathrm{a}}^{2+}\right)},$$

where *τ*_react,PLC_ is a function of the PLC enzymatic activity regulated by calcium and *τ*_coll,PIP2_ a function describing Gqα–PLC collisions with PIP_2_:

8 $${\tau }_{\mathrm{c}\mathrm{o}\mathrm{l}\mathrm{l},{\mathrm{P}\mathrm{I}\mathrm{P}}_{2}}\left(t\right)=\frac{{S}_{\mathrm{m}\mathrm{v}}}{{\alpha }_{3}{D}_{{\mathrm{P}\mathrm{I}\mathrm{P}}_{2}}\left[{\mathrm{P}\mathrm{I}\mathrm{P}}_{2,\mathrm{t}\mathrm{o}\mathrm{t}}-{\mathrm{P}\mathrm{I}\mathrm{P}}_{2,\mathrm{u}\mathrm{s}\mathrm{e}\mathrm{d}}(t)\right]},$$

where *α*_3_ is the PIP_2_–PLC collision factor, *D*_PIP2_ the diffusion rate for PIP_2_, PIP_2,tot_ the total number of PIP_2_ and PIP_2,used_(*t*) the number of accumulated DAG molecules.

DAG lifetime is limited by the activity of DAG kinase (DGK), which increases as cytosolic Ca^2+^ concentration rises:

9 $${\tau }_{\mathrm{D}\mathrm{A}\mathrm{G}}\left({\mathrm{C}\mathrm{a}}^{2+}\right)={\tau }_{\mathrm{D}, \mathrm{d}\mathrm{a}\mathrm{r}\mathrm{k}}{e}^{-{\beta }_{4}{A}_{\mathrm{D}\mathrm{G}\mathrm{K}}\left({\mathrm{C}\mathrm{a}}^{2+}\right)},$$

where *τ*_D,dark_ is the time constant for the decay of DAG in dark, *A*_DGK_ a function describing the calcium dependence of DGK activity, and *β*_4,_ an activation constant for the action of DGK on DAG.

Accumulation of DAG leads to the opening of light-activated channels. This process was modelled using the Monod–Wyman–Changeux allosteric transitions model. The number of open channels was presented as the product between the number of channels in the active state *N*_act_ and the probability of a channel being in the open state:

10 $${N}_{\mathrm{o}\mathrm{p}\mathrm{e}\mathrm{n}}\left(t\right)={N}_{\mathrm{a}\mathrm{c}\mathrm{t}}\left(t\right)\frac{{(1+{K}_{\mathrm{O}}\left[\mathrm{D}\mathrm{A}\mathrm{G}\mathrm{d}\right])}^{n}}{{(1+{K}_{\mathrm{O}}\left[\mathrm{D}\mathrm{A}\mathrm{G}\mathrm{d}\right])}^{n}+{(1+{K}_{\mathrm{C}}\left[\mathrm{D}\mathrm{A}\mathrm{G}\mathrm{d}\right])}^{n}/{Y}_{0}\left({\mathrm{C}\mathrm{a}}^{2+}\right)},$$

where [DAGd] = [DAG(*t*–*τ*_DAGdelay_)], *K*_O_ and *K*_C_ ligand (DAG) binding affinity parameters for the open and closed channel protein conformations, respectively; *Y*_0_ is the probability of a channel opening in the dark; *n* = 4 is the number of channel subunits forming a functional channel.

The current through the channels consists of currents of Ca^2+^, Na^+^, Mg^2+^ and K^+^ and can be described using the Goldman–Hodgkin–Katz equation:

11 $${I}_{\mathrm{T}\mathrm{R}\mathrm{P},q}\left(t\right)={N}_{open}\left(t\right){w}_{q}{P}_{1}{z}_{q}F{\beta }_{q}{V}_{m}\frac{{{C}_{q,\mathrm{i}\mathrm{n}}\left(t\right)-C}_{q,\mathrm{o}\mathrm{u}\mathrm{t}}\left(t\right){e}^{-{\beta }_{q}{V}_{m}}}{1-{e}^{-{\beta }_{q}{V}_{m}}},$$

where *P*_1_ is permeability of an open channel, *N*_open_*w*_*q*_*P*_1_ = *P*_*q*_ the permeability of open channels to ion species *q*, *z*_*q*_ its valence and *V*_*m*_ membrane potential. *β*_*q*_ = *z*_*q*_*F*/*RT*, where *F* is Faraday constant, *R* gas constant and *T* temperature. *C*_*q,*in_ and *C*_*q,*out_ are the microvillar and outside concentrations of ion *q*, respectively. *P*_*q*_(*t*) can be obtained from the total permeability of microvillus membrane if permeability ratios for different ions are known.

The influx of calcium inactivates metarhodopsin, stopping generation of activated Gq proteins. As DAG is degraded by DGK, TRP channels close, aided by activation of PKC.

#### Modifications to the model

The model was modified with the goal of accelerating bump generation and globally speeding up its kinetics. Although several strategies of parameter tuning could be utilized to achieve these goals, we made as few changes to the model parameters in the most physiological way as possible. The changes are described below and are also listed separately in Table 2.

**Table 2:** A full list of changes to the original *D. melanogaster* QB model (Nikolic et al. 2010) to produce the *P. americana* QB

Parameter	Definition	Value in *D. mel*	Value in *P. am*	Justification and the effect of changes
*w*_Ca_	Ca^2+^ permeability	0.877	0.675	The changes reflect the putative *P. americana* TRPL to TRP channel expression ratio of 90:10 and yield increased QB
*w*_Mg_	Mg^2+^ permeability	0.101	0.010	
*w*_Na_	Na^+^ permeability	0.011	0.315	
*w*_K_	K^+^ permeability	0.011	0	
*P*_1_	Permeability of an open TRP channel	1.0	2.7	Increases the total permeability to account for high unitary conductance of TRPL
*I*_calx,sat_	Saturation current for the *Calx* pumps	12 pA	8 pA	Decreased to reduce interference with QB current
*τ*_DAGdelay_	Time constant for DAG to activate TRP	12 ms	7 ms	Decreases mean latency
*τ*_P,dark_	Time constant for the decay of Gqα-PLC in dark	100 ms	40 ms	These two parameters were altered to prevent reopening of transduction channels due to the persisting elevated DAG
*τ*_Ddark_	Time constant for the decay of DAG in dark	80 ms	40 ms	
*τ*_GDP_	Time for GDP–GTP exchange	5 ms	2 ms	Decreases mean latency
*D*_G_	Diffusion constant of Gq protein	1.2 µm^2^ s^−1^	3 µm^2^ s^−1^ 6 µm^2^ s^−1^	Decreases mean latency
*D*_Gα_	Diffusion constant of Gqα subunit	1.5 µm^2^ s^−1^	4 µm^2^ s^−1^ 8 µm^2^ s^−1^	Decreases mean latency

In addition to these changes, as a part of modelling experiments, we varied Gq protein diffusion coefficients, and the numbers of Gq, PLC, and PIP_2_ as described in “Results”

First, ionic permeability of the microvillar membrane was altered to reflect the predominant expression of TRPL channels in the *P. americana* retina (Immonen et al. 2014; Saari et al. 2017). These channels are less selective for Ca^2+^ than TRP channels. To model the cockroach channel permeability, we used a TRPL to TRP channel expression ratio of 90:10; the Ca^2+^ to Na^+^ permeability ratio P_Ca_:P_Na_ for TRPL of 4:1, and for TRP of 100:1; and the TRPL to TRP single channel conductance ratio of 35:8. As a result, the total selectivity of the light-activated conductance to Ca^2+^ decreased from 0.877 to 0.675, the total selectivity to Na^+^ increased from 0.011 to 0.315, and selectivity to Mg^2+^ decreased from 0.10 to 0.01. Permeability to K^+^ was reduced to zero. These changes alone increased the current bump amplitude threefold, due to larger Na^+^ current. The total channel permeability *P*_1_ was increased by 2.7 times to account for the relatively high TRPL conductance and to match the amplitude of the experimentally derived current bump. In addition, the “saturation current for the *Calx* pumps” was lowered to 8 pA to decrease its contribution to the quantum bump current.

Our previous analysis suggested that the kinetics of the cockroach current bump in vivo should be much faster than the current bumps recorded in vitro and faster than the current bump in *D. melanogaster* (Fig. 5a) (Ignatova et al. 2019; Nikolic et al. 2010). Therefore, we altered the model to accelerate the bump kinetics. This included the reduction of the empirical “time constant for DAG to activate TRP” from 12 to 7 ms, which altered the mean latency accordingly and accelerated the second phase of the quantum bump onset.

Two parameters were altered to prevent reopening of the channels: “time constant for the decay of GPLC* in dark” was lowered from 100 to 40 ms, and “time constant for the decay of DAG in dark” was decreased from 80 to 40 ms. To reduce mean latency, we lowered “time for GDP–GTP exchange” from 5 to 2 ms and the “time constant for DAG to activate TRP” as mentioned above. Also, as a part of the “default cockroach QB” we increased Gq protein diffusion coefficients from 1.2 to 3 µm^2^ s^−1^ for the trimeric Gq protein, and from 1.5 to 4 µm^2^ s^−1^ for the α-subunit of Gq protein. This led to a decreased mean QB latency.

Finally, we changed the threshold of bump amplitude detection so that only bumps with absolute amplitudes above 5 pA were counted. The latency measurement threshold was changed dynamically so that on average mean latencies were determined at 10% of average peak amplitude for consistency with the 10% amplitude threshold used for latency measurement in our experiments.

### Data and statistical analysis

Quantum bumps were extracted and analysed using a custom Matlab script (by Paulus Saari) using template-matching algorithm. At the initial stage of statistical analysis, the Shapiro–Wilk normality test was applied to data samples to determine if they could be analysed using parametric statistical methods. Data in the samples that did not pass the normality test are presented in figures using box plots and compared using Mann–Whitney *U* test (MWUT). The samples that passed the normality test were analysed with parametric statistical methods as indicated. Such data are presented as mean ± s.d. and compared using a two-tailed unpaired *t *test with unequal variances. Spearman’s rank order correlation coefficient (SROCC, ρ) was used in analyses of correlations. In figures, (*) indicates *P* < 0.05 and (**) stands for *P* < 0.01. Throughout the text (*n*) stands for experimental group size.

## Results

### *P. americana PLC* and *Gqα* genes and RNA interference

**Fig. 1:**
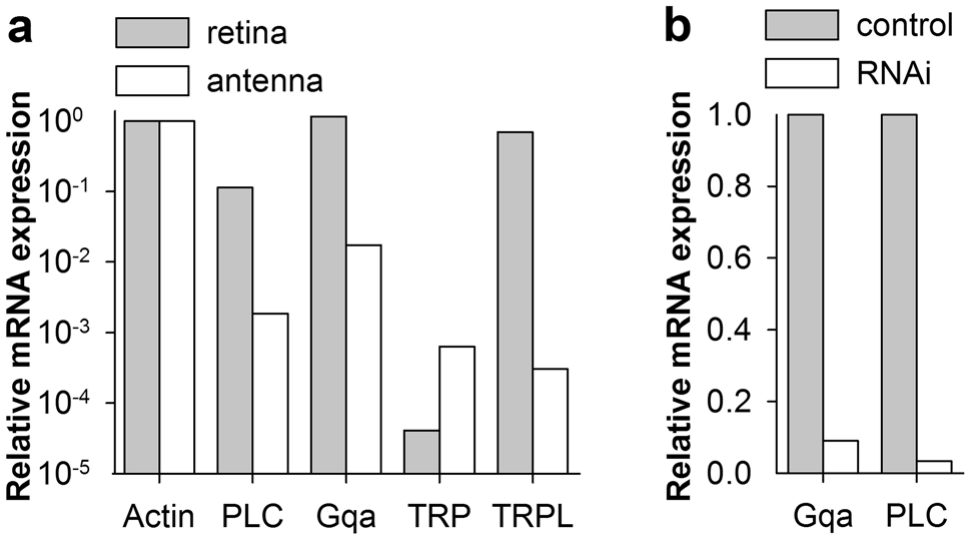
Relative abundances of mRNA in the transcriptomes. **a** Comparison of expression of four phototransduction-related genes (Gqα, PLC, TRP and TRPL) in the retinal and antennal transcriptomes; the concentrations of actin mRNA were used as references. **b** dsRNA induced downregulation of Gqα and PLC mRNA expression in *P. americana* retina. RT-qPCR results show large decrease in the relative expression levels of both Gqα and PLC transcripts 21 days after injection

The phosphoinositide-specific phospholipase C (PLC, GenBank accession number MN443916) found in *P. americana* retina codes a 1095 amino acid protein that has 65% sequence similarity with the protein encoded by *norpA* gene in *D. melanogaster* (accession number NP_001162661.1) The gene that codes the 353 amino acid α-subunit of guanine nucleotide-binding protein Gq (Gqα, accession number MN443915) in *P. americana* retina has 86% sequence identity with the α-subunit of *D. melanogaster* Gqα, isoform D (accession number NP_725196.1). Both of these genes are highly expressed in the cockroach retinal transcriptome with abundance levels close to actin. Figure 1a compares relative abundances of Gqα, PLC, TRP and TRPL mRNA in the retinal and antennal transcriptomes. The levels of actin were used as the reference.

Separate experiments using long double-stranded RNA to knockdown the PLC and Gqα genes (Table 1) in the cockroach retina strongly decreased the expression of the target transcripts when compared to sham-injected retinas as analysed with RT-qPCR (Fig. 1b).

### Minor effects of knockdowns on QB properties

We focused our analysis not only on quantum bumps but also evaluated other important parameters, including membrane capacitance (*C*_m_), membrane resistance at resting potential (*R*_m_) (the membrane time constant, the product of *C*_m_ and *R*_m_, sets the low-pass filtering properties of the membrane and thus influences the voltage bump kinetics), and absolute sensitivity.

Quantum bumps were evoked by 1 ms flashes of light applied once per second. Stimulus intensity was adjusted to evoke single bump responses with a probability < 0.7. Voltage responses apparently containing more than one bump were excluded. Bump latency was measured as the interval between the stimulus and the moment the bump voltage reached 10% of its maximum value.

**Fig. 2:**
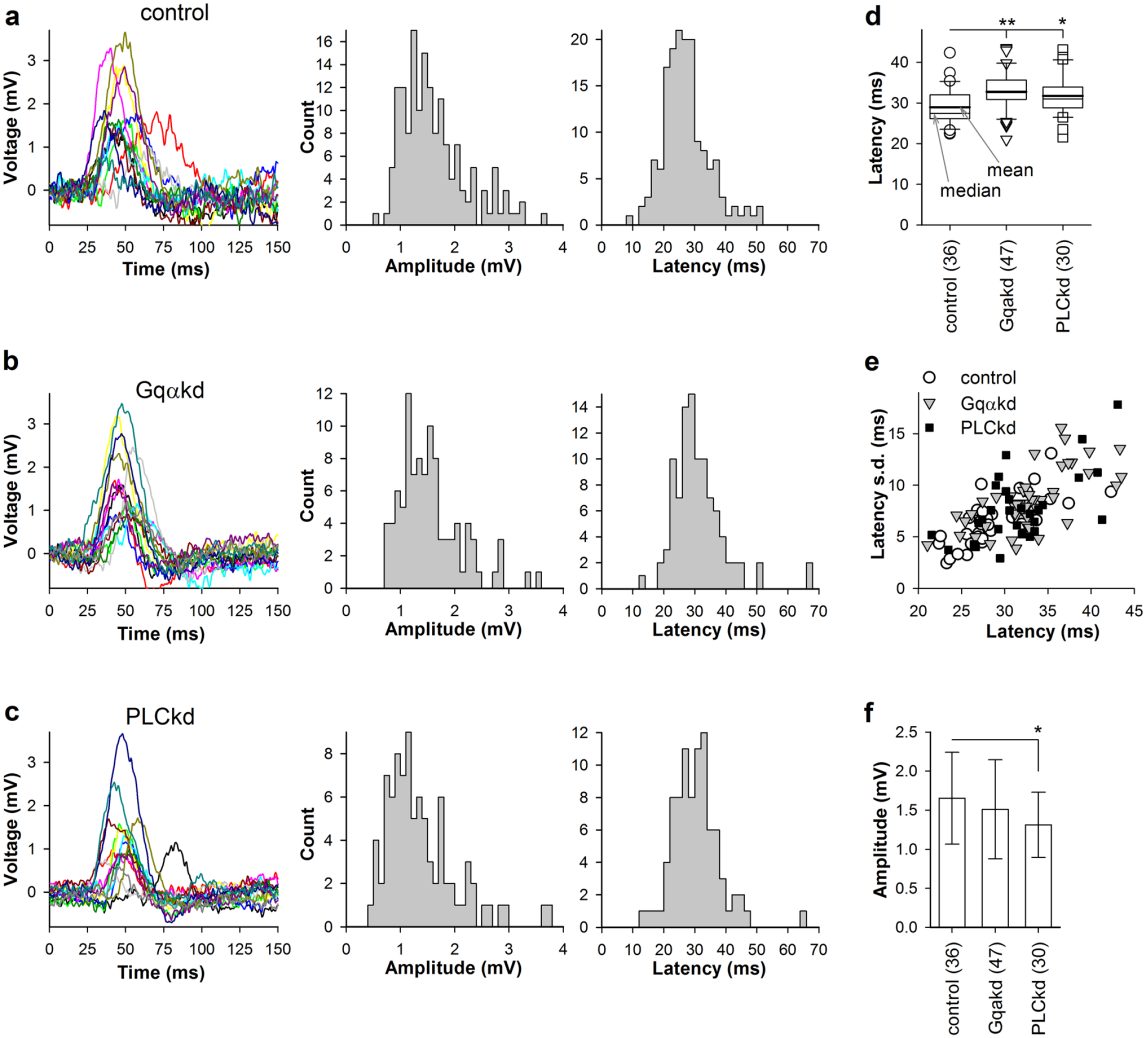
Voltage bumps in control, Gqα and PLC knockdown photoreceptors. **a**–**c** Representative examples of voltage bumps evoked in control (**a**), Gqαkd (**b**) and PLCkd (**c**) photoreceptors. Bumps were evoked by low-intensity 1 ms pulses eliciting responses with 10–60% probability; 15 bump traces are shown for each cell. Centre panels show distributions of all bump amplitudes and right panels show latency distributions for the same cells (see “Results” for details). **d** Box plot of mean latencies. The comparison of control and Gqαkd latencies gave *P* = 0.00044; the comparison of control and PLCkd values yielded *P* = 0.012, MWUT; in this and all other figures the numbers in parentheses indicate the number of cells; in this and all other box plots, thick horizontal lines in the boxes designate means; thin lines designate medians. **e** Scatter plot for mean latencies and associated standard deviations. **f** Comparison of group-average bump amplitudes; unpaired *t* test was used for statistics (*P* = 0.009 for the comparison between control and PLCkd mean bump amplitudes)

Figure 2a–c shows typical recordings of bumps from control, Gqα and PLC knockdown (Gqαkd and PLCkd, respectively) photoreceptors. Left panels demonstrate 15 bumps each as they were elicited, without alignment. Middle panels show amplitude distributions, and right panels demonstrate latency distributions for the same photoreceptors. Note that the amplitude distributions are somewhat distorted because bumps with amplitudes < 0.5 mV could not be reliably isolated from noise and thus such small bump-like events were not used in the analysis. The box plot in Fig. 2d compares group-average and median latency values for all three groups. We presented the data in the form of a box plot because the distribution of mean latencies in control did not pass the normality test. Mean bump latencies increased by ~ 10% in Gqαkd and PLCkd photoreceptors compared to control. Figure 2e shows scatter plots of latency dispersion measures (s.d.) against mean latencies. Although all three plots overlap notably, the control data concentrate closer to the origin than the data from Gqα and PLC knockdowns. Comparison of group-average amplitudes suggested a slight decrease of the quantum bump amplitude in PLCkd cockroaches (Fig. 2f).

### Changes in membrane resistance, capacitance and absolute sensitivity

**Fig. 3:**
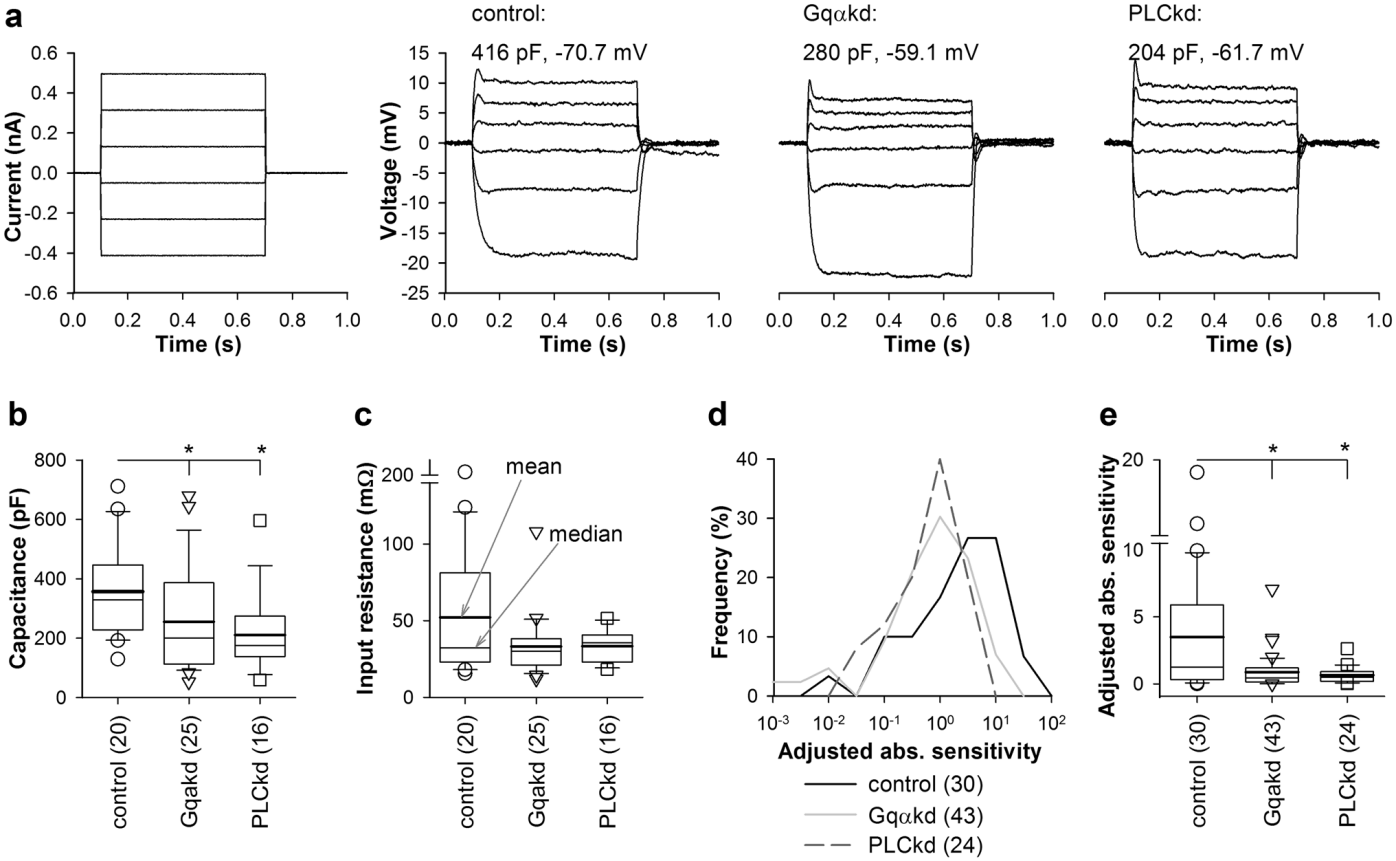
Changes in *R*_m_, *C*_m_ and absolute sensitivity. **a**, **b***C*_m_ was smaller in Gqα and PLC knockdown than control photoreceptors. **a** A current injection protocol and representative voltage responses of dark-adapted control, Gqαkd and PLCkd photoreceptors. The three photoreceptors shown here had similar input resistance *R*_m_ (compare response amplitudes to hyperpolarizing current steps) but different *C*_m_ values. The *C*_m_ can be inferred from the different onset kinetics: the control photoreceptor was characterized by the slowest onsets of the voltage responses, indicative of relatively high time constants and, accordingly, of high *C*_m_ as indicated; resting potential values (shown above the voltage traces) were subtracted from the recordings. **b** Box plot comparing *C*_m_ values shows that they are statistically significantly different (control vs. Gqαkd *P* = 0.019; and control vs. PLCkd *P* = 0.0025, MWUT). **c** Box plot of *R*_m_ values. **d** Distributions of adjusted absolute sensitivities for each photoreceptor were obtained in the following way. First, the bump probability was divided by the stimulus intensity and ND filter level, yielding an absolute sensitivity value. Next, all values were normalized to the level at which the median sensitivity in control was found and histograms were obtained. **e** Box plot compares adjusted absolute sensitivities. These values were statistically significantly different (control vs. Gqαkd *P* = 0.009; control vs. PLCkd *P* = 0.014, MWUT)

Similar to the previous studies involving RNAi of important photoreceptor proteins (Immonen et al. 2017; Saari et al. 2017), we found changes in the photoreceptors of Gq and PLC knockdowns, which could not be directly linked to altered gene expression. We, therefore, refer to such changes as non-specific compensations. These included decreased group-average membrane capacitance, lowered membrane resistance at resting potential (also known as input resistance), absolute sensitivities, and altered resting potentials (Fig. 3). Figure 3a shows typical voltage responses to step current injections in the dark. Figure 3b compares mean *C*_m_ values. *C*_m_ was significantly smaller in Gqαkd and PLCkd than in control photoreceptors. Similarly, input resistances were somewhat smaller in both Gqαkd and PLCkd retinas in comparison to control, albeit the group differences were not statistically significant (Fig. 3c).

Absolute sensitivity was measured in the following way: because bumps were evoked at different light intensities in different cells, to enable their comparison, we recalculated bump rates for a common light intensity. The original bump probabilities were first divided by the stimulus intensity and then normalized to the level at which the median sensitivity in control was found. For example, if bumps were recorded from a photoreceptor in a control animal with probability 0.4 at ND3, and stimulus intensity was 5 (5 mA of LED driving current), we obtain 0.4/10^–3^/5 = 80, whereas if bumps were recorded from a Gqαkd photoreceptor with probability 0.5 at ND2 (one decade brighter than ND3) and at stimulus intensity of 2, we obtain 0.5/10^–2^/2 = 25. The difference renders the latter cell ~ 3 times less sensitive than the former. After all sensitivities were calculated, all numbers were then divided by a factor corresponding to the ND level at which the median absolute sensitivity in control was found. This gave adjusted absolute sensitivities (Fig. 3d, e). The differences in adjusted absolute sensitivity between cells can be interpreted as the differences in the number of bumps that can be evoked by a stimulus of the same intensity and duration. In a less sensitive photoreceptor, the number of bumps and the adjusted absolute sensitivity are smaller; in a more sensitive photoreceptor, they are larger.

Absolute sensitivity was strongly reduced in both groups of the knockdown photoreceptors (Fig. 3d, e). Figure 3d shows that the distributions of adjusted absolute sensitivities for photoreceptors from Gqαkd and PLCkd were shifted negatively relative to control, and Fig. 3e shows a box plot comparison of adjusted absolute sensitivities. On average, the absolute sensitivity was four times lower in Gqαkd and 5.4 times lower in PLCkd than in control.

Finally, group-average resting potentials were more positive in the knockdown than in the control photoreceptors. The resting potential was − 65.3 ± 8.5 mV (*n* = 36) in control vs. − 58.2 ± 12.5 mV (*n* = 47) in Gqαkd (*P* = 0.003 for comparison with control, unpaired *t *test), and − 58.8 ± 12.7 mV (*n* = 30) in PLCkd (*P* = 0.015 for comparison with control, unpaired *t *test).

**Fig. 4:**
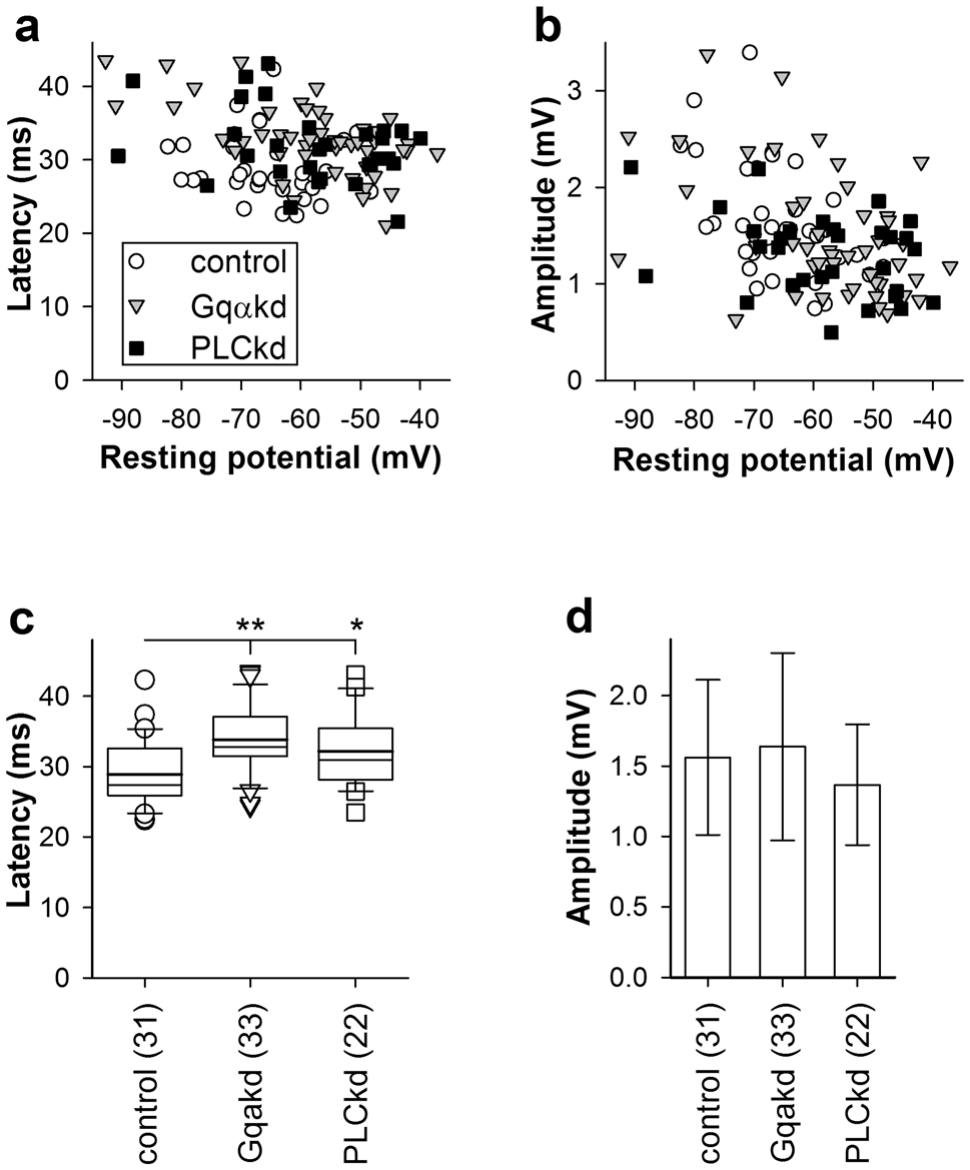
Voltage bump properties and resting potential. **a**, **b** Dependencies of voltage bump latencies (**a**) and amplitudes (**b**) on resting potential; all correlations were statistically significant (see “Results”). To account for the differences in the average resting potential between the groups, the groups were modified in the following way. Several cells characterized by the least negative resting potentials in Gqαkd and PLCkd groups, and by the most negative resting potentials in the control group were excluded; the resulting group-average values were – 63.5 ± 11.0, – 63.4 ± 11.1, and – 63.3 ± 7.3 mV, respectively. **c** Latencies for the modified groups using MWUT were statistically significant. **d** Group-average bump amplitudes for the modified groups were not statistically significant

When we examined relationships between the voltage bump parameters and resting potential, two correlations were found. The first was an unexpected correlation between resting potential and mean latency (Fig. 4a). The SROCC values were − 0.32 (*P* = 0.058, *n* = 36) for control, − 0.51 (*P* = 0.0002, *n* = 47) for Gqαkd,  − 0.23 (*P* = 0.22, *n* = 30) for PLCkd. We tested if changing resting potential with a continuous current injection of either polarity could alter mean latency but found no difference (data not shown).

The second correlation was between the mean voltage bump amplitude and resting potential (Fig. 4b). The SROCC values were − 0.50 (*P* = 0.0026, *n* = 36) for control, − 0.44 (*P* = 0.0022, *n* = 47) for Gqαkd, and − 0.26 (*P* = 0.16, *n* = 30) for PLCkd. This correlation was anticipated because voltage bump amplitude depends both on the driving force for the light-induced current and the membrane resistance, which generally decreases with depolarization.

Considering these correlations, proper comparison of voltage bump properties required the exclusion of some data from the experimental groups to minimize the influence of the differing group-average resting potentials. This was achieved by removing from the control group the cells characterized by the most negative resting potentials, and from the knockdown groups the cells characterized by the most positive resting potentials, until the group-average resting potentials were almost identical (see legend to Fig. 4). While the differences in latency between the control and knockdown groups remained statistically significant (Fig. 4c), the differences in mean amplitudes were no longer significant (Fig. 4d).

### QB latency, amplitude, and quantum efficiency in the model

Changes in quantum bump statistics observed in Gqαkd and PLCkd photoreceptors were surprisingly minor despite a dramatic suppression of gene expression (Fig. 1). Reasons for such small changes could be (1) slow turnover of Gqα and PLC, (2) other related proteins take over the functions of the reduced ones and (3) compensatory homeostatic changes in the rhabdomere could allow redistribution of Gq and PLC molecules among a smaller number of microvilli, thus maintaining high densities. Assuming that membrane thickness does not change in the knockdowns compared to control, the last scenario, involving a decrease in the number of microvilli and thus membrane area, would manifest in a decreased membrane capacitance and decreased absolute sensitivity, which is consistent with our findings. However, the absolute sensitivity measurements described above did not allow us to distinguish between a loss of sensitivity caused by a hypothetical decrease in the number of sampling units and those due to the failure of the phototransduction cascade to generate a quantum bump after the successful activation of a rhodopsin receptor by a photon.

**Fig. 5:**
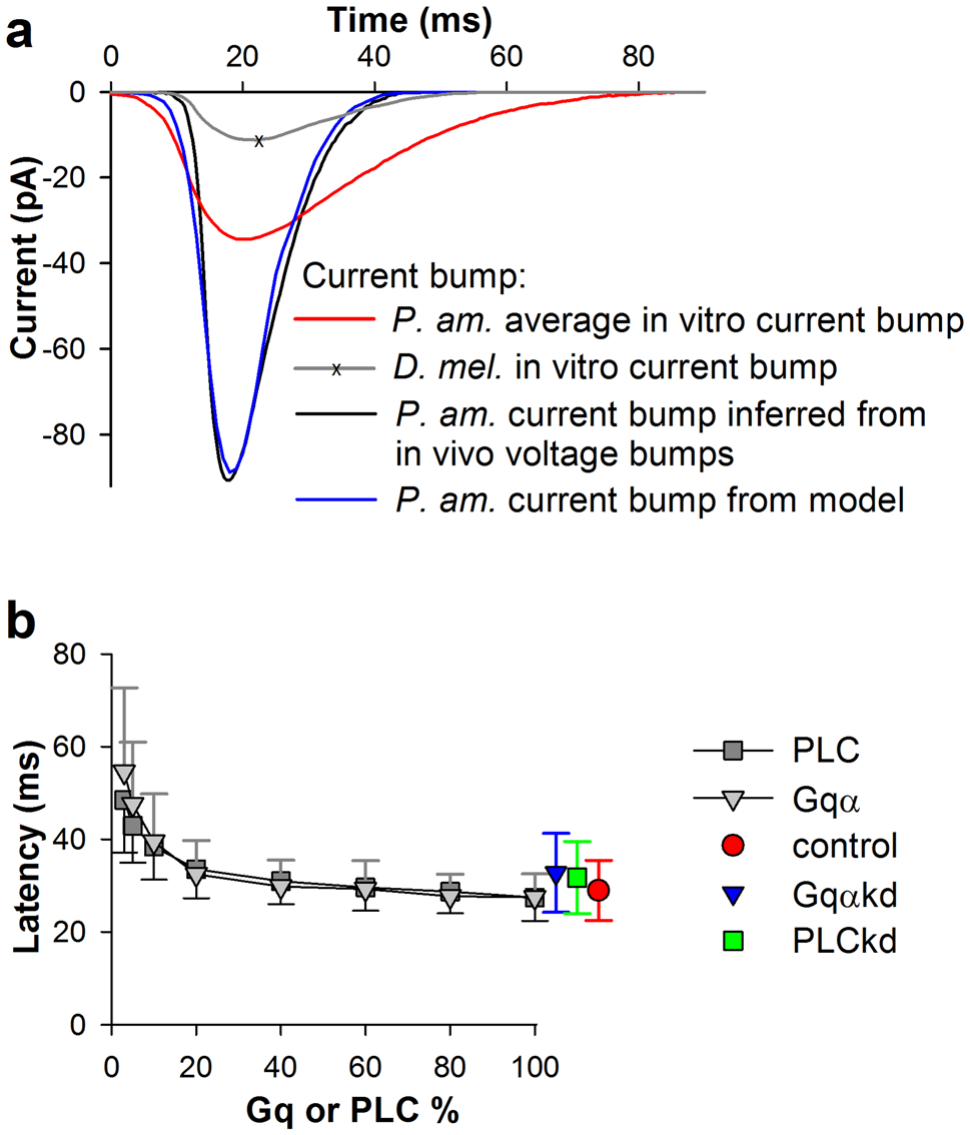
Model of *P. americana* current bump and effect of reduction of Gqα and PLC on bump latency and amplitude. **a** Group-average current bumps obtained in patch-clamp experiments in *P. americana* (red) (Ignatova et al. 2019) and *D. melanogaster* (grey) (Nikolic et al. 2010) are superimposed with a current bump *derived* by fitting from the group-average voltage bump in vivo (black, see description in (Ignatova et al. 2019)), and with a bump obtained using our model (blue); bumps were positioned so that their peaks coincide; the cockroach current bump amplitudes (experimental bumps were recorded at a holding potential of – 82 mV) were adjusted for proper comparison with the average *D. melanogaster* bump (recorded at a holding potential of – 70 mV) using a reversal potential value of + 10 mV. **b** Dependencies of mean bump latencies on concentration of Gqα and PLC proteins in experiments and simulations. The measured latency values for control and knockdown photoreceptors are also indicated. In these simulations, we used the diffusion rates for Gqαβγ and Gqα of 3 and 4 µm^2^ s^−1^, respectively; in this figure and Fig. 6, each value is an average of parameters obtained from bumps evoked in 200 trials; the number of evoked bumps depended on the quantum efficiency and varied from ca. 190 (95%) under the close to “normal” conditions to ca. 90 (45%) under the most extreme modelling situations (see Fig. 6e)

Quantum efficiency is the probability of eliciting a quantum bump after activation of rhodopsin. To investigate how changes in the levels of Gq and PLC could influence quantum efficiency, quantum bump latency, and amplitude, we developed a model of the cockroach quantum bumps based on a previously published stochastic model for *D. melanogaster* (Nikolic et al. 2010). We assumed that there are no major differences between phototransduction cascades in *D. melanogaster* and *P. americana*. The modifications to the original model are described in “Methods” and Table 2. This model allowed us to generate a mean current bump with kinetics similar to those of the previously *inferred* (from simulations) *P. americana* current bump (Ignatova et al. 2019), and with the mean latency matching our in vivo group-average control results (Fig. 5a). Figure 5b shows how changes in Gq or PLC concentrations affected the mean latency and its spread in the simulations. Surprisingly, significant increases in mean latency were observed only when the protein amounts were reduced by ~ 80%.

**Fig. 6:**
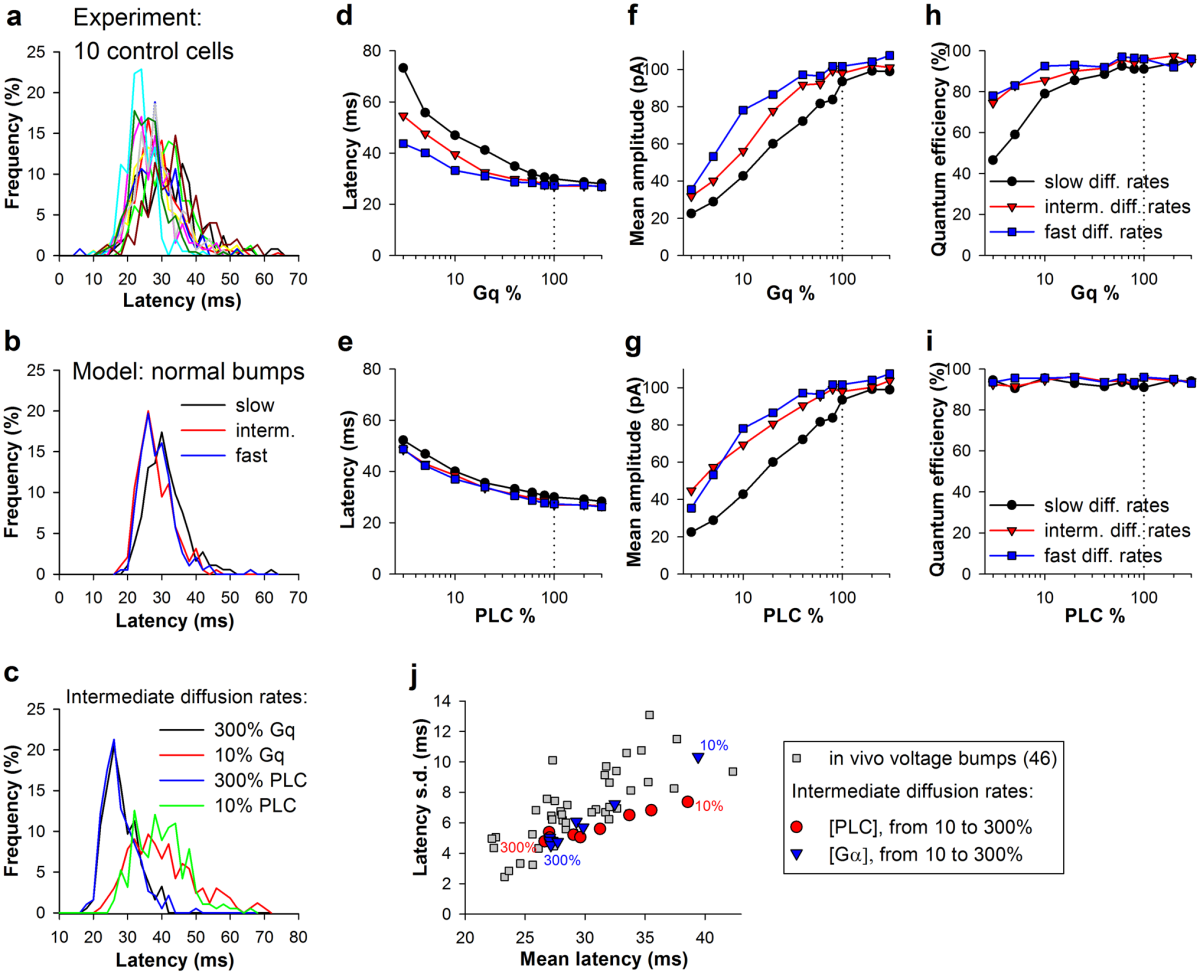
Effects of Gq diffusion, Gqα and PLC abundances on quantum bumps in the simulated model. **a** Normalized quantum bump latency distributions for ten photoreceptors in control; numbers of bumps varied from 122 to 311. **b** Latency distributions for “normal” simulated bumps obtained using different Gq protein diffusion rates. Three pairs of diffusion coefficients for Gqαβγ and Gqα were used: correspondingly, 1.2 and 1.5 µm^2^ s^−1^ (“slow diffusion rates”), 3 and 4 µm^2^ s^−1^ (“intermediate diffusion rates”), and 6 and 8 µm^2^ s^−1^ (“fast diffusion rates”). **c** Changes in latency distributions associated with either threefold increase or tenfold decrease in the number of Gq and PLC; simulations were performed at the intermediate Gq protein diffusion rates. **d**, **e** Effects of Gqα diffusion rates and Gqα (**d**) and PLC (**e**) abundances on mean bump latency. **f**, **g** Effects of Gqα diffusion rates and Gqα (**f**) and PLC (**g**) abundances on mean bump amplitude. **h**, **i** Effects of Gqα diffusion rates and Gqα (**h**) and PLC (**i**) abundances on quantum efficiency; quantum efficiency was calculated as a probability of producing a quantum bump after conversion of a rhodopsin into metarhodopsin. **j** Correlations between mean latencies and latency s.d. values for the experimental bumps in control and simulated bumps following changes in Gq and PLC abundances; simulations were performed at the intermediate Gq protein diffusion rates; the numbers indicate protein concentrations for the extreme data points

The durations of three overlapping stages can be affected by changes in the levels of Gq and PLC. The first consists of the diffusion of a Gqαβγ to the metarhodopsin and the formation of Gqα and is presumably limited by the number of Gq complexes in the microvillus and their effective diffusion speeds. The second stage involves diffusion of the Gqα to a PLC and their binding; it depends on the availability of PLC molecules and the Gqα diffusion coefficient. The third stage involves breakdown of PIP_2_ by the Gqα–PLC complex; it lasts until the opening of light-activated channels and is mainly determined by the diffusion of PIP_2_ to the activated Gqα–PLC and the sensitivity of the channels to the PIP_2_ breakdown/accumulation of DAG. Therefore, we expanded our simulations to investigate how changes in diffusion coefficients for Gqαβγ, Gqα, PIP_2_, and the concentration of PIP_2_ could influence the quantum bump properties (Fig. 6).

First, we studied how mean latency, mean amplitude and quantum efficiency could depend on the diffusion coefficients for Gqαβγ and Gqα. In the original model, the relatively low coefficients of 1.2 and 1.5 µm^2^ s^−1^ were used, respectively (Lamb and Pugh Jr 1992; Nikolic et al. 2010). However, the Gq protein moves freely under the plane of membrane. It is a peripheral protein complex attached to the membrane lipids at two points (Zhang et al. 2004), so that its diffusion may be more similar to the diffusion of the membrane lipids rather than integral proteins. According to Ramadurai et al. (2009), diffusion coefficients for membrane lipids and integral proteins at the protein densities in the microvillar membrane in the range of ~ 5000 proteins/µm^−2^ [estimated from (Kumar and Ready 1995; Paulsen and Schwemer 1979; Schwemer and Henning 1984)] should be ~ 10 µm^2^ s^−1^ and ~ 3 µm^2^ s^−1^, respectively. In the model, we used three pairs of diffusion coefficients for Gqαβγ and Gqα: correspondingly, 1.2 and 1.5 µm^2^ s^−1^ as in the original model (“slow diffusion rate” in Fig. 6), 3 and 4 µm^2^ s^−1^ for most of the analysis (including data in Fig. 5; “intermediate diffusion rate” in Fig. 6), and 6 and 8 µm^2^ s^−1^ for the relatively fast diffusion rates (“fast diffusion rate” in Fig. 6).

First, we evaluated changes in quantum bump latency distributions. Figure 6a shows normalized latency distributions for ten representative photoreceptors in control and Fig. 6b distributions of latencies obtained in simulations using three different pairs of diffusion coefficients under otherwise normal conditions (no changes in Gq or PLC levels). The influence of Gq protein diffusion speed on latency is minor. This contrasts with changes in Gq or PLC concentrations, which resulted in significant changes in the shapes and means of latency distributions (Fig. 6c).

Simulation results shown in Fig. 6d, e indicate that the rates of Gqαβγ and Gqα diffusion have little effect on mean latency when the concentrations of Gq protein are relatively high. Faster diffusion noticeably compensated for the loss of the protein only when Gq concentration fell below 30% (Fig. 6d). In contrast, the Gqαβγ and Gqα diffusion rates had almost no effect on the dependence of mean latency on PLC concentration over its entire range (Fig. 6e). The effects of Gqα and PLC depletion on the mean bump amplitude were similar, with faster diffusion linked to bigger bumps and vice versa (Fig. 6f, g). Next, we examined changes in quantum efficiency. It depended strongly on both Gqα concentration and diffusion rates (Fig. 6h), but did not depend on PLC concentration at all (Fig. 6i). Figure 6j shows correlations between mean latencies and measures of latency dispersion for the experimental bumps in control and simulated bumps after changes in Gq and PLC levels.

**Fig. 7:**
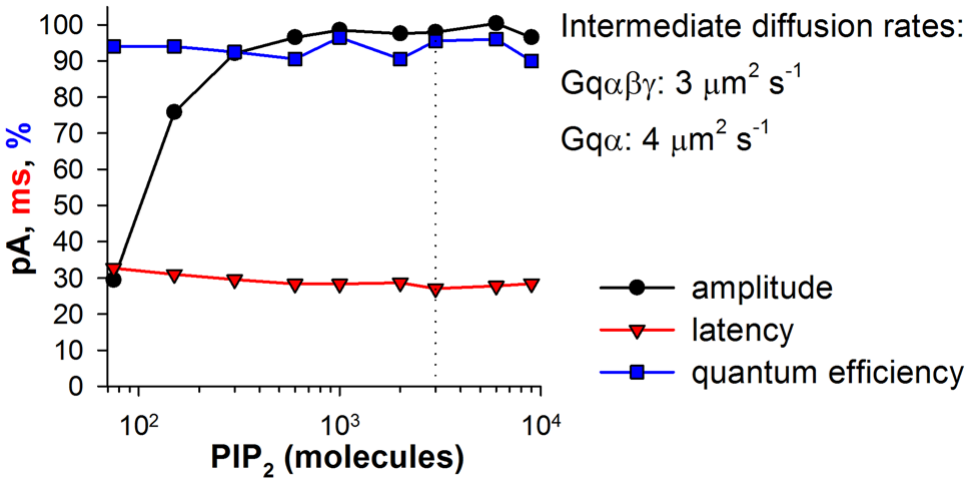
Effects of PIP_2_ level on quantum bump efficiency, mean latency, and mean amplitude at the intermediate Gq diffusion rates

Finally, we tested how PIP_2_ availability affects the quantum bump (Fig. 7). Reduction in PIP_2_ concentration had much smaller effects on the quantum bump properties than the reductions in the levels of Gqα and PLC.

## Discussion

We found that silencing *P. americana Gqα* and *PLC* genes by RNAi resulted in small changes in the photoreceptor responses to single photons despite large suppressions of *Gqα* and *PLC* transcripts in the retina. Group-average latencies were prolonged in both knockdowns by ~ 10%, whereas group-average bump amplitudes did not decrease significantly. These results differ substantially from the previous findings in *D. melanogaster* photoreceptors, where mutations or transcriptional silencing that drastically reduced the Gq and PLC levels, decreased the amplitude of quantum bumps and increased their latency (Hardie et al. 2002). We also found that the membrane input resistance, membrane capacitance and absolute sensitivity of both knockdowns were significantly altered.

Our modelling results were consistent with the experimental observations, suggesting that expression levels of Gq and PLC in the normal retina may be redundant. We hypothesize that excessive expression of these genes may be necessary to improve the speed, amplification and quantum efficiency (in case of Gq, see below) of the phototransduction cascade.

Our model suggested that a decreased Gqα but not PLC concentration is associated with reduced efficiency of the cascade (Fig. 6h, i). Although decreased quantum efficiency is consistent with our findings of the relatively low absolute sensitivities in both knockdowns, and also with the previous findings in *D. melanogaster* (Hardie et al. 2002), this effect cannot be separated from the loss of absolute sensitivity associated with what we consider the non-specific changes in the photoreceptor, especially with the reduced *C*_m_ [the relations between absolute sensitivity and *C*_m_ are discussed at length in Frolov et al. (2018)]. These presumably compensatory changes are similar to those documented previously in other cockroach retinal protein knockdown experiments (Immonen et al. 2017; Saari et al. 2017). Interestingly, increased ionic permeability of photoreceptor membrane at rest was also described in ion channel mutants in *D. melanogaster* (Vähäsöyrinki et al. 2006), suggesting the presence of intrinsic mechanisms that upregulate leak conductance(s) to substitute for a missing ion channel. (No comparable information is available regarding changes in *C*_m_). However, Gq and PLC are not ion channels, and so the decreased resistance (Fig. 3c) has no obvious explanation.

From the visual ecological perspective, high quantum efficiency is a highly desirable trait for nocturnal species, where each photon captured in the dark can carry a lot of information and thus is valuable. In contrast, diurnal insects whose facets in daylight are bombarded by millions of photons per second may not need high quantum efficiency, because each transduced photon elicits a very small voltage response, and thus necessarily carries little information. Thus, depending on the lifestyle and behaviour, evolutionary pressure to develop high quantum efficiency may vary.

Our results indicate that having many Gq and PLC proteins in the microvillus can noticeably shorten the mean latency. While the latency itself may not be important for signal transfer, unless it is so prolonged that it limits the reaction speed, the intrinsic latency dispersion, with its standard deviation being a linear function of mean photoreceptor latency (Figs. 2e, 6j), is important (Ignatova et al. 2019). The noise arising from the variability in the timing of quantum bump latency is one of the three components of phototransduction noise in microvillar photoreceptors (the other two are due to the variabilities in quantum bump amplitude and duration). (There is also a transducer-related component of noise due to variability in the duration of the microvillus refractory period (Song et al. 2012) but it is unknown if it depends on Gq and PLC levels.) Latency variability contributes to the broadening of multi-photon “impulse” responses with consecutive loss of higher frequency resolution (Ignatova et al. 2019; Lillywhite and Laughlin 1979). It follows from the dependence of latency dispersion on mean latency that reducing the former by shortening the latter could be one strategy to improve detection and transmission of fast visual signals. Thus, deficiencies in Gq and PLC associated with increased mean latency could result in a narrower signalling bandwidth than in wild-type animals.

What mechanisms could decrease the latency of a microvillus during a single-photon response? The factors influencing the latency include the densities of Gq and PLC proteins, Gqαβγ and Gqα diffusion constants, GDP to GTP replacement rate on the activated Gqα, the rate of PIP_2_ breakdown by PLC, and the sensitivity of light-activated channels to PIP_2_ breakdown. All protein-related factors can probably be altered either by changes in gene expression or tissue environment, e.g. both PLC activity and TRP sensitivity depend on the level of Ca^2+^ in the microvillus (Nikolic et al. 2010). However, the diffusion constants may be less amenable to functional regulation than the protein-related parameters.

What could be other causes for the surprisingly minor effects of the knockdowns? First, it is possible that the turnover rates of Gq and PLC are relatively low, and these proteins could be present in high concentrations 3 weeks after dsRNA injections. We did not measure the actual Gq and PLC protein levels in the knockdown retinas and the mRNA concentrations were investigated in retinas of groups of seven–nine animals rather than in individual retinas that were used in electrophysiological experiments. However, the relative mRNA expression levels were very low. Previously, knockdown of *P. americana* TRPL channels using similar methods as described here, dramatically reduced the quantum bump amplitudes within 7–20 days after injection (Immonen et al 2017; Saari et al 2017). Second, the large changes of membrane capacitance and absolute sensitivity in the knockdown photoreceptors suggest extensive remodelling of the rhabdomeres. While such remodelling could be associated with a decrease in the number of microvilli, the individual microvilli may still have adequate amount of these proteins to produce nearly normal responses to light.

In conclusion, we found two types of changes in the knockdown photoreceptors. Putative homeostatic compensations manifested in the reduced group-average membrane capacitances, suggesting decreased membrane areas, and reduced absolute sensitivities in both groups relative to control. The specific changes at the level of single-photon responses were surprisingly minor: despite > tenfold suppression of mRNA levels, quantum bump amplitudes were not altered significantly, but their latencies increased by ~ 10% in both knockdowns. Modelling yielded non-linear dependencies of latency on the Gqα and PLC concentrations, suggesting that expression of these proteins in the microvillus may be highly redundant for generation of quantum bumps. However, we did not test if the proposed redundancy could affect signal transfer by graded voltage responses in brighter light.

## Abbreviations

*C*_m_: Membrane capacitance, in picofarads (pF)
DAG: Diacylglycerol
dsRNA: Double-stranded RNA
GDP: Guanosine diphosphate
GTP: Guanosine-5′-triphosphate
Gq: Phototransduction G-protein
IP_3_: Inositol trisphosphate
LIC: Light-induced current
ND: Neutral density filter
PCR: Polymerase chain reaction
PIP_2_: Phosphatidylinositol 4,5-bisphosphate
PKC: Protein kinase C
PLC: Phospholipase C
QB: Quantum bump
*R*_m_: Membrane resistance
TRP: Transient receptor potential channel
TRPL: Transient receptor potential-like channel
